# Identification of Key Genes Driving Tumor Associated Macrophage Migration and Polarization Based on Immune Fingerprints of Lung Adenocarcinoma

**DOI:** 10.3389/fcell.2021.751800

**Published:** 2021-11-04

**Authors:** Jing Wu, Jiawei Zhou, Qian Xu, Ruth Foley, Jianqiang Guo, Xin Zhang, Chang Tian, Min Mu, Yingru Xing, Yafeng Liu, Xueqin Wang, Dong Hu

**Affiliations:** ^1^School of Medicine, Anhui University of Science and Technology, Huainan, China; ^2^Anhui Province Engineering Laboratory of Occupational Health and Safety, Anhui University of Science and Technology, Huainan, China; ^3^Key Laboratory of Industrial Dust Prevention and Control & Occupational Safety and Health of the Ministry of Education, Anhui University of Science and Technology, Huainan, China; ^4^The Charles Institute of Dermatology, School of Medicine, University College Dublin, Dublin, Ireland; ^5^Affiliated Cancer Hospital, Anhui University of Science and Technology, Huainan, China

**Keywords:** lung adenocarcinoma, tumor infiltrating cells, tumor microenvironment, clinical prognosis, macrophage polarization

## Abstract

The identification of reliable indicators in the tumor microenvironment (TME) is critical for tumor prognosis. Tumor associated macrophages (TAMs) are the major component of non-tumor stromal cells in TME and have increasingly been recognized as a predictive biomarker for lung adenocarcinoma (LUAD) prognosis. Here, we report the development of a prognosis model for LUAD using three immune-related genes (IRGs) detected in The Cancer Genome Atlas (TCGA) which potentially regulate TAMs in TME. In 497 LUAD patients, higher immune scores conferred better overall survival (OS). We identified 93 hub IRGs out of 234 for further prognostic significance. Among them, three IRGs (BTK, Cd1c, and S100P) were proved to be closely correlated to the prognosis of patients with LUAD. Moreover, the immune risk score (IRS) based on the gene expression level of the three IRGs was an independent prognostic factor for OS. Higher IRS predicted lower OS, higher mortality and worse tumor stage. With a good predictive ability [area under the ROC curve (AUC) in TCGA = 0.701, AUC in GEO = 0.722], the IRS contributed to a good risk stratification ability of the nomogram. Immunologically, the three IRGs were related to M1 macrophages and NK cell subsets in TME. Interestingly, by characterizing these immune components *in situ* we found that S100P is a driver for tumor cells to induce TAM migration and M2 polarization in the immunosuppressive tumor niche. We identified the key genes driving TAM migration and transformation and elucidated the immune landscape of LUAD. The data suggest that IRGs from TME have the potential to become indicators for estimating cancer prognosis and guiding individualized treatment.

## Introduction

Lung adenocarcinoma (LUAD) is one of the leading causes of cancer-related deaths globally due to early metastasis and poor prognosis (Skoulidis et al., 2018). An effective and accurate method for prognosis prediction is urgently needed. Immunosuppressive cells in the tumor microenvironment (TME) facilitate tumor metastasis and tumor stem cell formation, which are closely related to clinically poor prognosis (Li et al., 2019). Recent studies have found that the status of TME was modulated by tumor infiltrating cell (TIC) types and immune-related genes (IRGs), during which the immune cells undergo transformation and dysfunction, particularly the polarization of macrophages towards M2 and suppression of CD8+ T cells and NK cells (Tuo et al., 2020; Zheng et al., 2020). Single cell sequencing is well suited to identify diverse immune cell subsets in TME, however, it costs too much for routine diagnostic and prognostic evaluation of LUAD patients (Guo et al., 2020). Biopsy specimens are not sufficient for high throughput analysis of TIC subsets. Therefore, it is necessary to identify crucial IRGs closely related to immune cell dysfunction for an economically acceptable and reliable prognostic prediction. A network analysis integrating the immune gene signature and cell map is important to illuminate the mechanism of TME development, which could ultimately improve the accuracy of prognosis prediction.

The TME is the cellular environment in which tumors are located. It is composed of immune cells, mesenchymal cells, endothelial cells, inflammatory mediators, cytokines, and extracellular matrix (ECM) (Lim and Ghajar, 2021). Immune cells and stromal cells are two main non-tumor components in TME. Understanding of the cross-talk between them is of great value for the diagnosis and prognosis of tumors (Hill et al., 2020). Apart from promoting tumor growth and progression, immunocompromised TICs are closely related to the poor clinical outcomes of targeted therapy, radiotherapy, and chemotherapy (Koliaraki et al., 2020). Tumor associated macrophages (TAMs) are the major component of tumor-infiltrating immune cells, and usually have a tumor-suppressing M1 phenotype or tumor-promoting M2 phenotype. After being tamed by tumor cells, TAMs were polarized to M2 with a phenotype of IL-10^*high*^, IL-12^*low*^, arginase-1^*high*^, CD206^*high*^, CD204^*high*^, and MHC-II^*low*^. M2 polarization promotes immunosuppressive tumor niche formation and is often associated with poor prognosis for patients, possibly due to the inhibited cytotoxicity of tumor killer cells. However, the mechanism of promoting M2 polarization by cross-talk between tumor cell and TIC remains unclear. The Cancer Genome Atlas (TCGA) and Gene Expression Omnibus (GEO) databases provide abundant data of differentially expressed genes (DEGs) in tumor tissues. Based on these DEGs, we have previously developed a prediction model for LUAD prognosis (Jiawei et al., 2020). However, most of these data come from the whole TME and fail to distinguish the source from tumor cells or immunocytes. To date, only few studies systematically explored the correlation between infiltrating immune cell population and the prognosis of LUAD patients. In view of the important role of tumor-associated immune cells in the TME status and prognosis, it is necessary to identify the key genes which can represent the immune status of TME and predict the prognosis of LUAD patients.

In this study, we identified S100P as a driver gene for tumor cells to recruit and polarize TAMs, a critical TIC subset for immunosuppressive tumor niche formation. We characterized the immune landscape of LUAD in which BTK, Cd1c, and S100P were highlighted as signature genes in both NK and TAM cells. The immune risk score (IRS) based on the three IRGs was identified as an independent predictive factor for LUAD prognosis. These findings may provide potential biomarkers for the diagnosis and prognostic prediction of LUAD, which is of great significance for understanding the mechanism of LUAD at the molecular level.

## Materials and Methods

### Raw Data Download

In this study, the RNA expression profile (GSE31210) data of LUAD was obtained from GEO public database.^1^ The selection criteria for the expression profile are as follows: (1) the detected samples are tissues, (2) all tissues are diagnosed as LUAD tissues and normal tissues, (3) the gene expression profile is mRNA, (4) samples collected from the same ethnic group, (5) the probes can be converted into the corresponding gene symbols, and (6) complete information analysis. The array data of GSE31210 includes 226 LUAD tumor tissues, including 20 cases where data on adjacent normal tissues was available. Subsequently, RNA sequencing data sets of 54 normal lung tissue cases and 497 LUAD cases and corresponding clinical data were downloaded from TCGA^2^ database.

### Estimated Scores of Immune and Stromal Tissues

ESTIMATE algorithm of R code version 3.6.2 was used to estimate the proportion of immune-stromal component in TME of each sample of LUAD in TCGA, exhibited in the form of three kinds of scores: Immune Score, Stromal Score, and ESTIMATE Score, which positively correlated with the ratio of immune component, stromal component and the sum of both, respectively (thus the higher the score, the larger the proportion of the corresponding component in TME).

### Survival Analysis

The survival R package was used to analyze the immune/stromal score and the survival rate of patients. A total of 458 tumor samples out of 497 had a detailed survival time record, with time span from 0 to 18.7 years, which were used for survival analysis. The survival curve was plotted by the Kaplan–Meier method with log-rank test; *p* < 0.05 was considered significant.

### Correlation Analysis Between Clinical Stages and Immune/Stromal Score

Clinicopathologic characteristics data corresponding to LUAD samples were downloaded from TCGA. R code was employed for analysis, and the Wilcoxon rank sum test was used to analyze the correlation between immune/stromal score and each clinical characteristic according to the number of clinical stages.

### Immune-Differentially Expressed Genes Between High-Score and Low-Score Groups Regarding Immune/Stromal Score

A total of 497 LUAD patients were categorized in high-score or low-score groups compared to the median, regarding immune score, and stromal score. Gene expression differential analysis was performed using the Limma in R software package and DEGs were generated by comparing high-score and low-score samples. Based on the immune genes in ImmPort database, the immune-DEGs in high-score and low-score samples were screened out. Screening condition: | log2(FC)| ≥ 1, *p* < 0.05.

### Kyoto Encyclopedia of Genes and Genomes Pathway and Gene Ontology Enrichment Analysis

Through Gene Ontology (GO) enrichment and Kyoto Encyclopedia of Genes and Genomes (KEGG) analysis, the biological functions of these immune-DEGs were comprehensively detected. Cellular component (CC), molecular function (MF), and biological process (BP) were selected in GO enrichment. All analysis used online was based on network of gene set analysis tool (WebGestalt^3^). Both the primary *p*-value and adjusted *p*-value (FDR) were less than 0.05, which was statistically significant.

### Protein–Protein Interaction Network Construction and Module Screening

The STRING database^4^ was used to detect protein–protein interaction (PPI) among all immune-DEGs. Cytoscape 3.6.1 software was used to build and visualize the PPI network. Molecular complex detection (MCODE) plug-in was used to screen out important modules and genes in the PPI network. Both MCODE score and node number greater than 5, and *p* < 0.05, were considered significant differences. Weighted correlation network analysis (WGCNA) was used to sort the modules of co-expressed genes in PPI network.

### Establishment of Prognostic Prediction Model

Univariate Cox regression analysis was performed on all key genes in the PPI network by using survival package in R, and candidate immune-DEGs related to prognosis were screened by log-rank test, and then further screened by LASSO regression analysis. Three IRGs were selected based on the correlation with prognosis from the core immune genes in the PPI network for further analysis as described below. Subsequently, based on the important candidate genes from the preliminary screening above, a risk regression model was constructed and the risk score was calculated to evaluate the prognosis of patients. The risk score of each sample was obtained according to the following formula:


$$R\,i\,s\,k\,s\,c\,o\,r\,e=\sum_{i=1}^{n}E\,x\,p\,i\,\beta \,i,$$

β, regression coefficient; Exp, gene expression value. To assess the performance of this prognostic model, LUAD patients were divided into low-risk and high-risk groups according to the median risk score. The risk score based on expression of three selected IRGs was named IRS. The difference of overall survival (OS) between the two groups was compared by log-rank test. Then, the prediction ability of the above model was evaluated using the survival ROC (receiver operating characteristic curve) package. In addition, 226 LUAD patient samples from GSE31210 data^5^ were acquired as validation samples to verify the predictive value of the prognostic model.

### Prognostic Value and Nomogram Construction of Different Clinical Features

The prognostic value of different clinical features of LUAD patients in TCGA and GSE31210 data sets was analyzed through univariate and multivariate Cox regression. ROC curve was used to verify the accuracy of different clinical features as independent prognostic factors in predicting the OS of LUAD patients. Then, the rms R package was utilized to draw a nomogram for predicting 3-year and 5-year OS of patients from the IRS based on expression of three selected IRGs, combined with other clinical features, and performed internal verification in the TCGA samples.

### Analysis of Immune Gene Expression and Clinical Characteristics

In order to study the correlation between the expression of three selected IRGs and patient survival and TNM (size of tumor, degree of regional lymph node involvement, and presence of metastasis) stage, we analyzed the expression level of each gene in LUAD tumor and adjacent cancer in the TCGA database, as well as the prognosis correlation, respectively, and used the network data GEPIA to verify the results. At the same time, we also analyzed the correlation between the expression of three genes and the clinical characteristics of LUAD patients.

### GSEA of Immune Genes

To study the regulatory mechanisms of three IRGs in LUAD patients, we divided LUAD patients into high-expression and low-expression groups according to the expression levels of three genes, and carried out GSEA.

### The Correlation Between Immune Genes and Tumor Infiltrating Cells

In order to study the correlation between three genes and TICs expression in LUAD patients, the CIBERSORT calculation method was utilized to estimate the TIC abundance distribution in all tumor samples. After quality filtration was performed, only 421 tumor samples with *p* < 0.05 were selected for the following analysis. The correlation between three genes and expression of prognostic immune cells was then analyzed by univariate analysis.

### Identification of Immune Genes and Prognosis-Related Tumor Infiltrating Cells in Clinical Specimens

Samples from patients with LUAD were selected to verify the expression of immune genes and prognostic TICs. All formalin-fixed and paraffin-embedded tissue samples were collected to detect the infiltration level of immune genes and prognostic immune cells. A total of 5 μm sections were taken from paraffin-embedded specimens, dewaxed and rehydrated. The tissue sections were then placed in EDTA antigen repair buffer (pH 8.0). After natural cooling, the glass slides were placed in PBS (pH 7.4) and shaken and washed on the decolorizing shaker for three times, each time for 5 min. Samples were incubated for 30 min with serum after hydrogen peroxide sealing. The primary antibody was added after appropriate dilution in PBS, and the slices were incubated at 4°C in a wet box overnight. After washing with PBS (pH 7.4) three times on the decolorizing shaker, the secondary antibody labeled with HRP of the corresponding species was added into the slides to cover tissues, and incubated for 50min at room temperature. The slides were washed in PBS (pH 7.4) for 5 min three times on the decolorizing shaker. When the slices were slightly dried, the CY3-TSA (GB1223, 1:2000; Servicebio) was added into the circle and incubated at room temperature for 10 min. The primary and secondary were removed by microwave treatment, and the second primary antibody and the corresponding HRP-labeled secondary antibody were added successively, then labeled with FITC-TSA (GB1222, 1:1000; Servicebio). Similarly, the slices were labeled by CY5. After DAPI re-staining of the nucleus, the film was sealed and photographed under microscope. All immunofluorescence (IF) sections were analyzed by ImageJ software. Primary antibodies were supplied and diluted as follows: CD68, GB14043, 1:1000; CD163, GB13340, 1:3000; CD16, GB14026, 1:1000; CD56, GB14041, 1:100; S100P, GB14147, 1:200 from Servicebio; BTK, A1576, 1:100 from Abclonal. CD68 and CD163 were used as markers for different types of TAMs, and CD16 and CD56 as NK cell markers. Nine visual fields were randomly selected in each section, and the correlation between immune cell infiltration and gene expression was analyzed by ImageJ.

### Primary Macrophage Culture and siRNA Transfection

To study the effect of S100P gene on the growth and polarization of macrophages in TME, we transfected siRNA_S100P into A549 cells for S100P gene knockdown. The siRNA_S100P was chemically synthesized by Gene Pharma Company (Shanghai, China), according to the sequence previously published by Camara et al. (2020), with sequence as follows: forward, 5-color AUGGAUGCCCAGGUGGGACTT-mur3′ and reverse-5′GUCCACCUGGCAUCUCCAUTTMU3′. In addition, the negative control siRNA NC was synthesized with scrambled sequences. A549 cells were transfected with siRNA_S100P and siRNA_NC for 12 h, and then co-cultured with PBMC-M (primary macrophages induced from human peripheral mononuclear cells) and THP-1 cells induced by PMA (180 ng/ml) for 48 h. All macrophages were identified by morphological characteristics and CD68 expression. Macrophage migration was detected by crystal violet staining in transwell incubation. Immunofluorescence (IF) was used to detect the transformation of macrophages to M1 and M2 after co-culture for 48 h. All the slides were analyzed by ImageJ software. The PBMC-M was induced as follows: (1) PBMC were isolated from human peripheral blood by discontinuous density gradient centrifugation on Ficoll-Isopaque. (2) PBMC were added into to 60 mm dishes and cultured in the incubator for 3 h. After the incubation, the supernatant was discarded, cells were washed slowly with PBS twice, and 2.5 ml DMEM was added followed by observation of the cells’ condition. (3) The cells were cultured with GM-CSF (100 ng/ml) for 7 days, changing half of the medium daily after the third day. THP-1 was induced by PMA as follows: (1) After THP-1 cells were treated with PMA (180 ng/ml) for 24 h, the cells were induced to differentiate into macrophages. (2) The cells were re-suspended with serum-free RPMI without irritants and PMA. The purity of the induced macrophages was identified by Giemsa staining and CD68 labeling.

## Results

### The Analysis Process of the Study

The analysis process was shown in Supplementary Figure 1. To estimate the proportion of TICs and the content of immune and stromal components in LUAD samples, transcriptome RNA sequence data of 551 patients were obtained from the TCGA database, and then calculated with the CIBERSORT and ESTIMATE algorithms, respectively. The PPI network was constructed, including immune genes differentially expressed between immune and stromal components. Next, three prognostic-related IRGs (BTK, Cd1c, and S100P) were obtained by univariate COX and lasso analysis on the core immune genes in the PPI network. The IRS calculated by the IRGs was used for relationship analysis. Clinical analysis includes survival rate and pathological features, and immunity analysis includes immunocyte populations by CIBERSORT and immune pathways by GSEA.

### The Correlation Between Immune and Stromal Score With Prognosis of Lung Adenocarcinoma Patients

**FIGURE 1:**
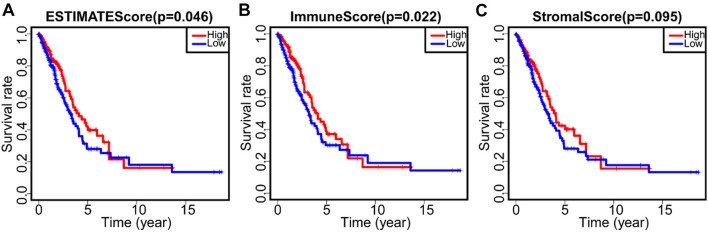
The correlation between score and survival rate of LUAD patients. **(A)** Kaplan–Meier survival curve of ESTIMATE score and survival rate of LUAD patients (*p* = 0.046). **(B)** Kaplan–Meier survival curve of immune score and survival rate of LUAD patients (*p* = 0.022). **(C)** Kaplan–Meier survival curve of stromal score and survival rate of LUAD patients (*p* = 0.095).

To calculate the correlation between the immune and stromal score with the survival rate of LUAD patients, Kaplan–Meier survival analyses on the immune score, stromal score, and ESTIMATE score were performed, respectively. The data showed that the higher the immune or stromal score, the more immune or stromal components in TME. ESTIMATE score was the sum of immune and stromal score, which represented the combined proportion of these two components in TME. As shown in Figure 1A, the proportion of combined components (immune components and stromal components) was positively correlated with OS rate. The immune score was positively correlated with survival rate (Figure 1B), while the stromal score was not significantly correlated with OS rate (Figure 1C). These data suggest that the immune components in TME could better reflect the prognosis of LUAD patients than all components.

### The Correlation Between Score and TNM Stage of LUAD Patients

To determine the relationship between the proportion of immune and stromal components and clinicopathological features, the clinical information of LUAD patients from TCGA database were considered. As shown in Supplementary Figure 2, the ESTIMATE score was negatively correlated with TNM stages (Supplementary Figure 2A, *p* = 0.0055), T grades (Supplementary Figure 2B, *p* = 0.0027), and M grades (Supplementary Figure 2D, *p* = 0.013). The immune score was also negatively correlated with TNM stages (Supplementary Figure 2E, *p* = 0.017), T grades (Supplementary Figure 2F, *p* = 0.00069) and M grades (Supplementary Figure 2H, *p* = 0.059) classification. Similarly, the stromal score was negatively correlated with stage (Supplementary Figure 2I, *p* = 0.0023), T grades (Supplementary Figure 2J, *p* = 0.017), and M grades (Supplementary Figure 2L, *p* = 0.0041). These results indicated that the proportion of immune and stromal components was related to the invasion and metastasis of LUAD, although N grades were not correlated to ESTIMATE score (Supplementary Figure 2C, *p* = 0.47), immune score (Supplementary Figure 2G, *p* = 0.58) and stromal score (Supplementary Figure 2K, *p* = 0.57).

### The Gene Ontology and Kyoto Encyclopedia of Genes and Genomes Functional Enrichment Analysis of Differentially Expressed Genes Shared by Immune and Stromal Components

**FIGURE 2:**
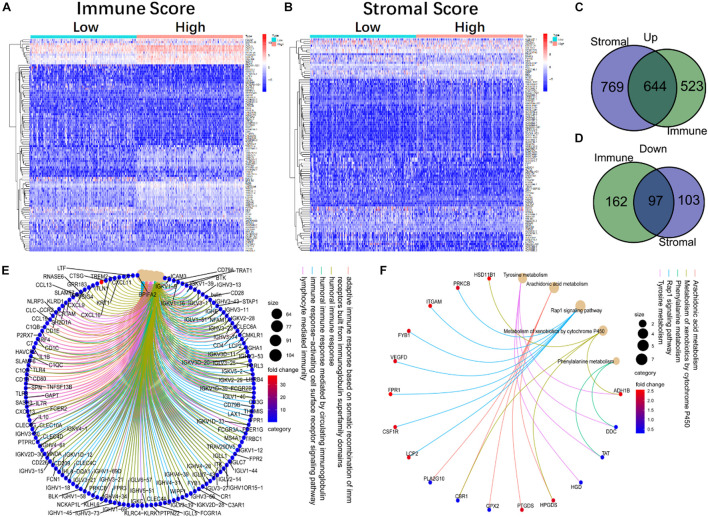
The heatmap, Venn diagram, GO, and KEGG enrichment analysis of DEGs in immune and stromal components. **(A)** The heatmap of DEGs in high and low immune score groups. **(B)** The heatmap of DEGs in the high and low stromal score groups. **(C,D)** The Venn diagram of up-regulated and down-regulated genes co-expressed in immune and stromal component. **(E,F)** The GO and KEGG enrichment analysis of 741 DEGs.

To confirm the exact changes related to immune and stromal components in TME, we compared gene expression of 458 tumor samples. A total of 1,426 DEGs from the immune components were achieved, among which 1,167 genes were up-regulated and 259 genes were down-regulated (Figure 2A). Similarly, 1,613 DEGs were obtained from stromal components, including 1,413 up-regulated genes and 200 down-regulated genes (Figure 2B). Next, we selected DEGs with same trend between the two components, and showed on Venn diagrams that 644 genes were up-regulated (Figure 2C), and 97 genes were down-regulated (Figure 2D) in both stromal and immune components. These 741 common DEGs were subjected to GO and KEGG enrichment analysis. Almost all the 741 DEGs were related to immune function, such as lymphocyte immunity and humoral immune response (Figure 2E), and these genes were enriched in Rap1 signaling pathway, drug metabolism, and cytochrome P450 metabolism (Figure 2F). These data suggested that the overall function of DEGs was closely related to immune activity, which means immune genes could be important parts of TME.

### The Analysis of Immune Genes and Protein–Protein Interaction Network

To further study the correlation between DEGs and immune function, 1,811 IRGs were taken from the ImmPort database and intersected with DEGs. The Venn diagram displayed a total of 234 immune genes (Supplementary Figure 3A). To further identify the key genes in the immune network, we imported these 234 IRGs into the STRING database, and used Cytoscape to visualize the PPI network and construct sub-networks. A total of 93 nodes and 961 edges were identified (Supplementary Figure 3B). The nodes represent the key proteins in the PPI network. The more edges a node had, the more important it was as a network hub. So, a total of 93 IRGs were identified from the PPI network. Then the MCODE tool was applied to process the co-expression network, identify possible key modules, and obtain a key module, including 31 nodes and 290 edges (Supplementary Figure 3C).

The functions of DEGs in key modules were mainly enriched in the following aspects: leukocyte proliferation, lymphocyte proliferation, monocyte proliferation, immune cell proliferation regulation, and some functions closely related to immunity. Among them, the gene with the largest number of connected nodes in the PPI network was IL-10 (Supplementary Figure 3D). To clarify the co-expression relationship between genes, we used WGCNA to analyze the co-expression modules of 234 network key genes and obtained three co-expression enrichment modules (Supplementary Figure 3E). In addition, we used WGCNA to analyze the co-expression of 93 network key genes and obtained two modules (Supplementary Figure 3F). We found that BTK, Cd1c and S100P belong to the same gene enrichment module, suggesting that three genes were co-expressed.

### Identification of Prognosis-Related Genes

**FIGURE 3:**
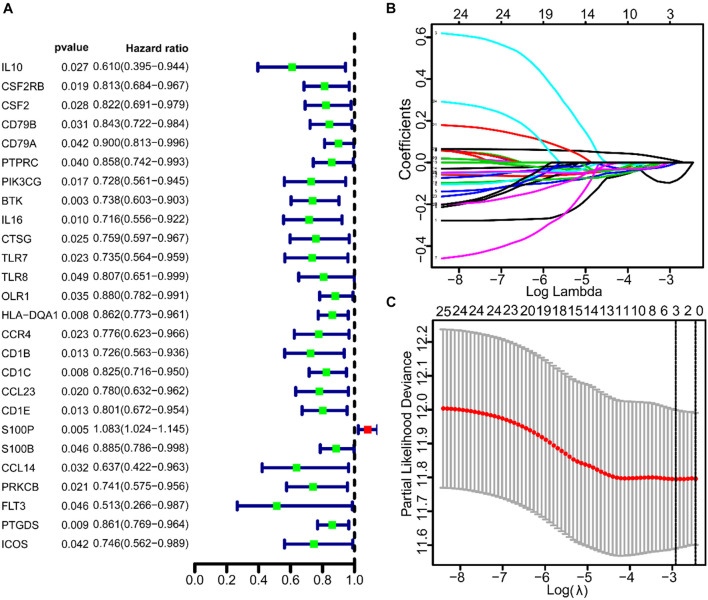
The univariate COX and lasso analysis of immune genes. **(A)** The univariate COX analysis of 93 IRGs. **(B,C)** The lasso analysis of 26 immune genes.

To study the prognostic significance of these genes, we performed univariate Cox regression analysis to assess the correlation between the expression levels of 93 IRGs and the survival rate of patients. Twenty-six prognosis-related IRGs were found (Figure 3A). Subsequently, to identify the key genes that were important in prognosis, we performed lasso analysis of these 26 prognosis-related IRGs. The resulting top three IRGs were identified as BTK, Cd1c, and S100P, and were found to correlate closely to the prognosis of LUAD patients (Figures 3B,C).

### Construction and Analysis of Immune Risk Score Model From Prognosis-Related Genes

To construct a prognostic model, we calculated the IRS based on the three IRGs (Table 1). The calculation formula is as follows:

**TABLE 1:** Top three prognosis-associated genes identified by lasso regression analysis.

**Gene symbol**	**Coefficient**	**HR**	**HR.95L**	**HR.95H**
BTK	–0.159	0.852	0.657	1.105
Cd1c	–0.082	0.921	0.77	1.101
S100P	0.049	1.05	0.989	1.115


IRS=(-0.15982×EXP⁢_⁢BTK)+(-0.08222×EXP⁢_⁢Cd1c)+(0.049485×EXP⁢_⁢S100⁢P).

**FIGURE 4:**
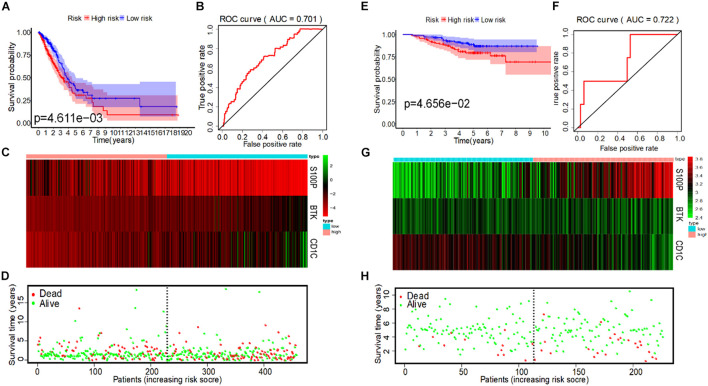
IRS analysis of prognosis models of 3 immune genes in TCGA and GSE31210 datasets. **(A)** The survival curves of low-risk and high-risk group in TCGA. **(B)** The ROC curve for predicting OS of patients based on the IRS to in TCGA. **(C)** The expression heatmap of three immune prognostic genes in the low-risk and high-risk group from TCGA. **(D)** IRS distribution and survival status of patients in the low-risk and high-risk group from TCGA. **(E)** The survival curve of low-risk and high-risk group in GSE31210. **(F)** The ROC curve for predicting OS of patients based on IRS in GSE31210. **(G)** The expression heatmap of three immune prognostic genes in the low-risk and high-risk group from GSE31210. **(H)** IRS distribution and survival status of patients in the low-risk and high-risk group from GSE31210.

To evaluate the predictive ability of the model, we divided the 458 LUAD patients from the TCGA into high-risk and low-risk groups for survival analysis based on the median of IRS (median = 0.997). The results showed that compared with the low-risk group, patients in the high-risk group had a poorer survival prognosis (Figure 4A). To further evaluate prognostic prediction ability of IRS, ROC analysis related to survival time was carried out. The area under the ROC curve (AUC) of the IRS model was 0.701 (Figure 4B), indicating a good performance in prognosis. Meanwhile, IRS was closely related to the expression levels of the three IRGs (Figure 4C) and the survival status of patients (Figure 4D). In addition, we selected the dataset of LUAD patients from GSE31210 for verification, and we found that IRS could also effectively predict survival rate (Figures 4E–H).

### The Prognostic Value of Different Clinical Features and the Construction of Nomogram

Currently, the clinical features of tumor patients are commonly used indicators for clinical evaluation of prognosis. Therefore, we used univariate and multivariate Cox regression to analyze the prognostic value of different clinical features of LUAD patients. The results from univariate COX analysis showed that tumor stage, primary tumor, lymph node metastasis and IRS were related to OS of LUAD patients (*p* < 0.001) (Table 2) in the TCGA. However, multivariate COX regression analysis results stated that tumor stage, lymph node metastasis (N) and IRS were only independent prognostic factors of OS (Table 2).

**TABLE 2:** The prognostic effect of clinical and molecular parameters in TCGA.

**Parameter**	**Univariate analysis**			**Multivariate analysis**		
	**HR**	**95% CI**	***p*-Value**	**HR**	**95% CI**	***p*-Value**
Age	1.011	0.994−1.028	0.18	1.014	0.997−1.031	0.102
Gender	1.07	0.777−1.474	0.676	0.82	0.586−1.148	0.248
Stage	1.671	1.441−1.938	< 0.001	1.302	1.027−1.652	0.029
T	1.599	1.305−1.957	< 0.001	1.248	0.999−1.599	0.051
N	1.84	1.522−2.225	< 0.001	1.398	1.071−1.825	0.014
IRS	2.512	1.523−4.124	< 0.001	2.451	1.426−4.212	< 0.001

**FIGURE 5:**
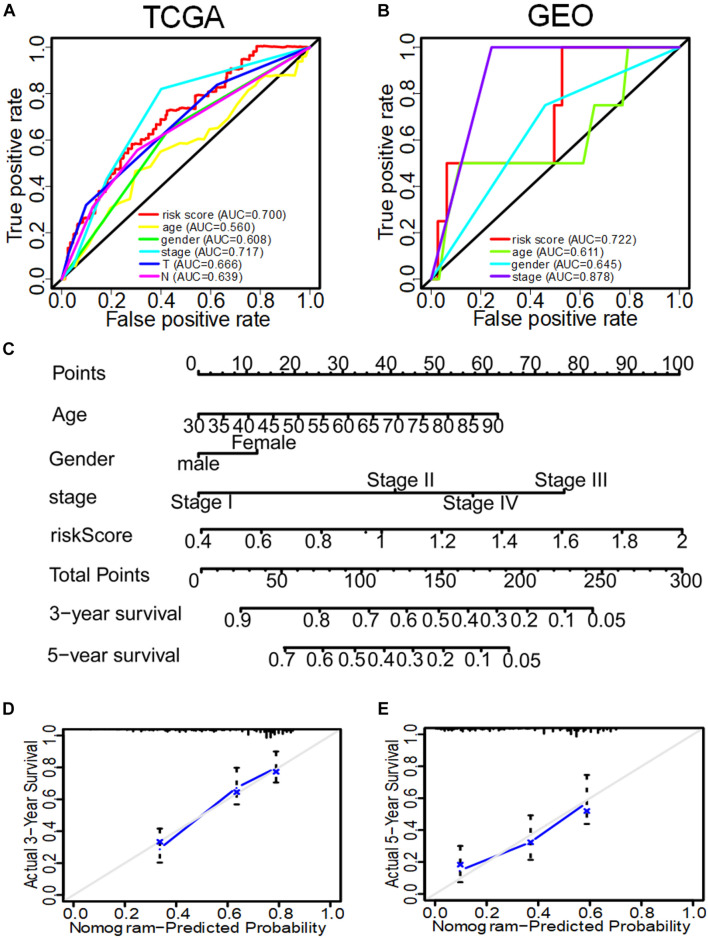
The ROC curve of evaluating the reliability of each clinical feature to predict the prognosis of patients in TCGA and GEO in and the nomogram of 3–5-year OS predicted by the IRS of three immune genes combined with other clinical features. **(A)** The ROC curve of each clinical feature to predict the prognosis of patients in TCGA. **(B)** The ROC curve of each clinical feature to predict the prognosis of patients in GEO. **(C)** The nomogram to predict the 3-year and 5-year OS of patients in TCGA. **(D)** The correction nomogram of predicting the 3-year OS of patients. **(E)** The correction nomogram of predicting the 5-year OS of patients.

In the GSE31210 dataset, univariate COX analysis found that stage and IRS were related to OS of LUAD patients (*p* < 0.001), while multivariate COX regression analysis found that stage and IRS were independent prognostic factors related to OS (Table 3). Subsequently, ROC curve was plotted to evaluate the reliability of each clinical feature to predict the prognosis of patients in TCGA and GSE31210 (Figures 5A,B). The area under the ROC curve of IRS was >0.7, predicting the prognosis of patients. The data indicate that IRS is an independent prognostic factor of OS. To visualize the predictive effect of each independent prognostic factor on OS, rms R package was employed to draw a nomogram of 3 to 5-year OS (Figure 5C). To evaluate the reliability of the nomogram, a correction chart was constructed and the 3-year and 5-year survival rate of LUAD patients was calculated by drawing the vertical line between the total point axis and each prognostic axis, showing a good consistency between the predicted result and the observed result (Figures 5D,E).

**TABLE 3:** The prognostic effect of clinical and molecular parameters in GEO.

**Parameter**	**Univariate analysis**			**Multivariate analysis**		
	**HR**	**95% CI**	***p*-Value**	**HR**	**95% CI**	***p*-Value**
Age	1.025	0.977−−1.075	0.306	1.035	0.985−1.083	0.181
Gender	1.519	0.780−2.955	0.219	1.482	0.572−2.366	0.676
Stage	4.232	2.175−8.236	< 0.001	3.904	1.966−7.752	0.001
IRS	1.928	1.107−3.357	< 0.001	1.484	1.810−2.719	< 0.001

### The Relationship Between the Three Immune-Related Genes and the Survival Time of Lung Adenocarcinoma Patients and TNM Stage

**FIGURE 6:**
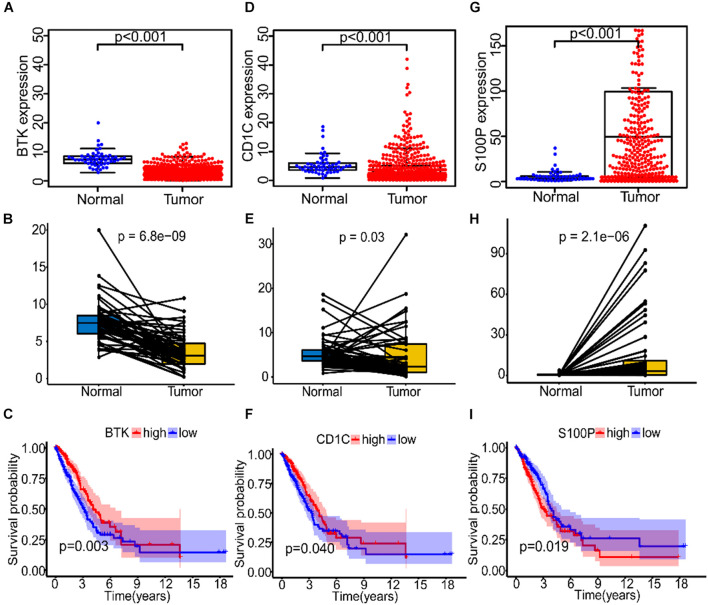
The expression and prognostic analysis of three immune genes in LUAD patients. **(A–C)** The correlation between the expression level of BTK in normal and tumor tissues and prognosis of patients. **(D–F)** The correlation between the expression level of Cd1c in normal and tumor tissues and prognosis of patients. **(G–I)** The correlation between the expression level of S100P in normal and tumor tissues and prognosis of patients.

To study the correlation between the expression of the three IRGs and survival rate of patients and TNM stage, we analyzed the expression level of each gene separately. In the TGGA, BTK had low expression in tumor tissues of LUAD patients (Figures 6A,B), and patients with high gene expression of BTK had significantly longer survival time (Figure 6C). Similarly, Cd1c was expressed at low levels in tumor tissues (Figures 6D,E), and LUAD patients with high gene expression of Cd1c survived longer (Figure 6F). On the contrary, S100P was highly expressed in tumor tissues (Figures 6G,H), and patients with low expression of S100P survived longer (Figure 6I).

Further, data in the GEPIA database were verified, and results confirmed the low expression of BTK and Cd1c in LUAD tumor tissues (Supplementary Figures 4A,C), and the finding that patients with high expression of BTK or Cd1c survived longer (Supplementary Figures 4B,D). S100P showed high expression in tumor tissues (Supplementary Figure 4E), but patients with low expression of S100P survived longer (Supplementary Figure 4F), which were consistent with the data from TGGA.

Further analysis showed that expression of BTK was negatively correlated with stage (Supplementary Figure 5A, *p* = 0.0071), T (Supplementary Figure 5B, *p* = 0.0018), and M classification (Supplementary Figure 5D, *p* = 0.036) of TNM stage, but there was no significant correlation with N classification (Supplementary Figure 5C, *p* = 0.3). Similarly, the expression of Cd1c was negatively correlated with stage (Supplementary Figure 5E, *p* = 0.005), T (Supplementary Figure 5F, *p* = 0.039), and M (Supplementary Figure 5H, *p* = 0.019) classification of TNM stage, but there was no significant correlation with N classification (Supplementary Figure 5G, *p* = 0.93). It is unexpected that there was no significant correlation between S100P and these clinical features (Supplementary Figures 5I–L).

### The Immune-Related Signaling Pathways Involving the Three Immune-Related Genes

To explore the immune-related signaling pathways involved in the three IRGs, we divided LUAD patients into high-expression and low-expression groups based on gene expression, and then GSEA was performed. The function of the high-expression group of BTK was mainly enriched in immune-related activities, such as autoimmunity, cell adhesion, chemotactic cytokines, and T cell receptors signaling pathway (Supplementary Figure 6A). The function of the low-expression group of BTK was mainly enriched in amino acid metabolism, base excision repair and some typical tumor pathways produced by proteins (Supplementary Figure 6B). The function of the high-expression group of Cd1c was mainly enriched in pathways related to immune function such as autoimmunity, B cell receptor signaling pathway, cell adhesion, cytokine receptor signaling pathway, and T cell receptor signaling pathway (Supplementary Figure 6C). The function of the low-expression group of Cd1c was mainly enriched in aminoacyl biosynthesis, cell cycle, P53 signaling pathway and RNA degradation pathway (Supplementary Figure 6D). The function of the high-expression group of S100P was mainly enriched in B cell receptor signaling pathway, cell adhesion molecule, cytokine receptor interaction, JAK_STAT_signaling pathway, and T cell receptor signaling pathway (Supplementary Figure 6E). The function of the low-expression group of S100P was mainly enriched in amino acid metabolism, base excision repair, cell cycle, sodium citrate cycle, and pathways related to polysaccharide biosynthesis (Supplementary Figure 6F). These results suggested that the three immune genes that we identified in this research may be a potential indicator of TME status.

### Tumor Infiltrating Cells Expression Profile in Lung Adenocarcinoma

**FIGURE 7:**
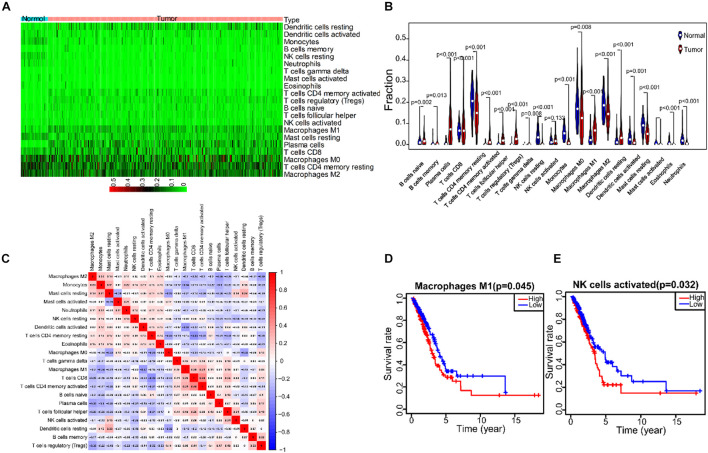
The correlation analysis between TICs expression profile in LUDA tumor tissue and prognostic. **(A)** The expression heatmap of 21 immune cells in LUAD tumor tissue and normal tissue. **(B)** The expression violin diagram of 21 immune cells in LUAD tumor tissue and normal tissue. **(C)** The correlation test of 21 immune cells expression. **(D)** The correlation between M1 macrophages and the prognosis of LUAD patients. **(E)** The correlation between NK cells activated and the prognosis of LUAD patients.

To describe the expression profile of immune cells in the TME, CIBERSORT was taken to analyze the TIC abundance distribution in all tumor samples. We found that there was a significant correlation between the gene expression profiles of at least one kind of immune cell subsets in 421 tumor samples (*p* < 0.05), and these samples were selected to construct the expression profile of 21 immune cells in LUAD (Figure 7A). The results of differential and correlation analysis showed that a total of 18 immune cells were differentially expressed. Among them, the cells with the highest amount in tumor tissues are M2 macrophages, M0 macrophages, M1 macrophages, CD4+ resting T cells and resting mast cells, which were all significantly more than those in normal tissue (Figure 7B). We also studied the correlation among the five cell subpopulations. M1 macrophages were negatively correlated with activated dendritic cells, monocytes and resting mast cells, and positively correlated with (γδ) T cells. Activated NK cells were negatively correlated with resting NK cells, monocytes and resting CD4 memory cells, and positively correlated with T follicular helper cells and resting mast cells (Figure 7C). Prognostic analysis showed that M1 macrophages and activated NK cells were closely related to prognosis of patients, and it was statistically significant (Figures 7D,E).

### Study of Three Immune-Related Genes and the Expression of Tumor Infiltrating Cells Related to Prognosis

**FIGURE 8:**
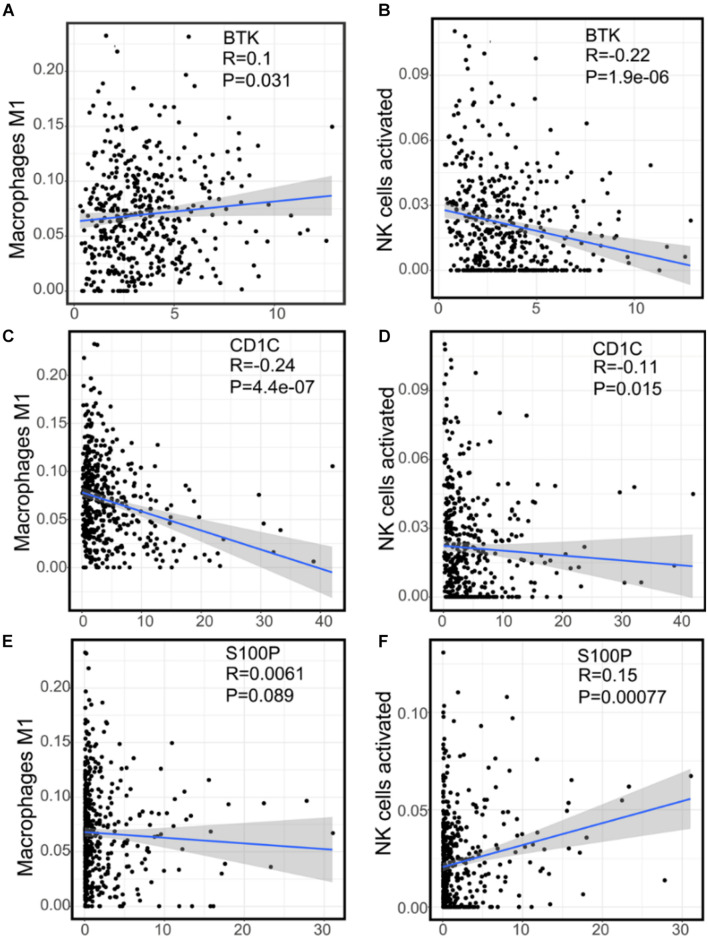
The correlation between the expression of three immune genes and two kinds of prognostic immune cells. **(A)** The correlation scatter plot of the expression of BTK and M1 macrophages. **(B)** The correlation scatter plot of the expression of BTK and NK cells activated. **(C)** The correlation scatter plot of the expression of Cd1c and M1 macrophages. **(D)** The correlation scatter plot of the expression of Cd1c and NK cells activated. **(E)** The correlation scatter plot of the expression of S100P and M1 macrophages. **(F)** The correlation scatter plot of the expression of S100P and NK cells activated.

To explore the correlation between the three IRGs and prognostic immune cell subgroups, we analyzed the expression level of each gene and the content of M1 macrophages and NK cell subgroup. The expression of BTK was positively correlated with M1 macrophages (Figure 8A, *p* = 0.031), and negatively correlated with activated NK cells (Figure 8B, *p* = 1.9e-06). Cd1c was negatively correlated with M1 macrophages (Figure 8C, *p* = 4.4e-07) and activated NK cells (Figure 8D, *p* = 0.015). S100P was not significantly correlated with M1 macrophages (Figure 8E, *p* = 0.089), but positively correlated with activated NK cells (Figure 8F, *p* = 0.00077).

### Verification of Immune-Related Gene Expression and Tumor Infiltrating Cells *in situ* Related to Prognosis

**FIGURE 9:**
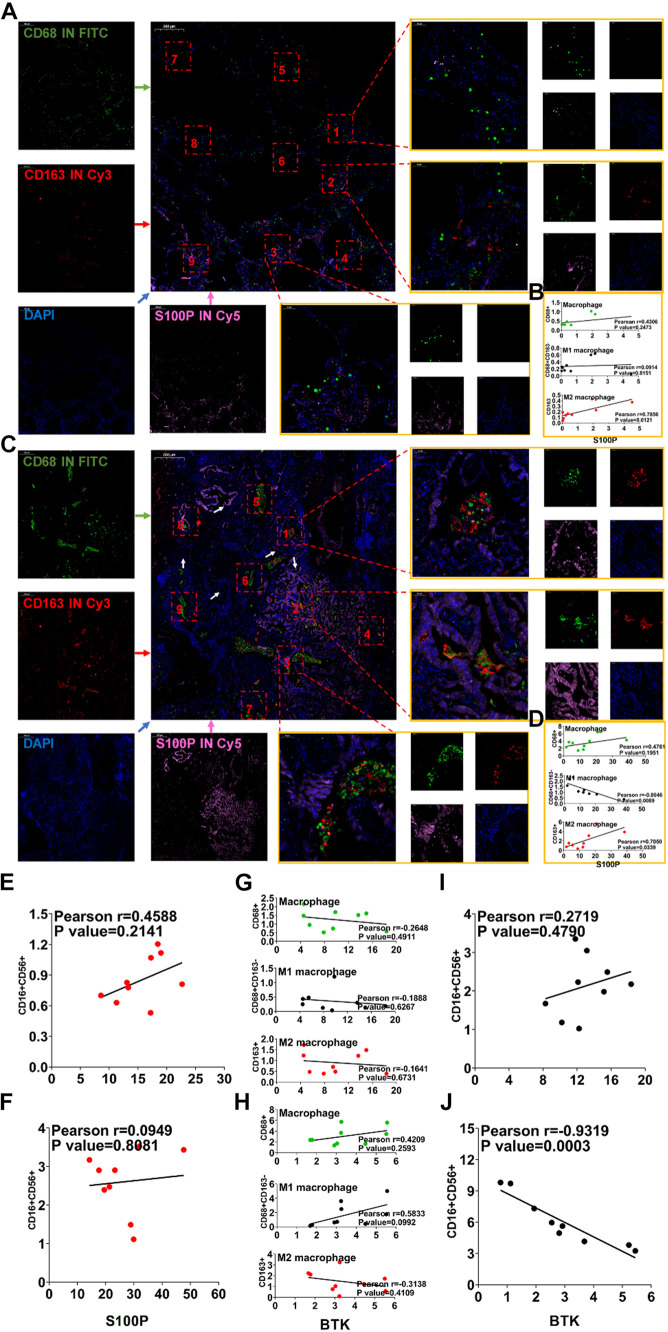
Immunofluorescence verification of IRG expression and TICs related to prognosis. **(A)** Immunofluorescence of S100P and macrophages in paracancerous tissues. **(B)** Correlation map of S100P and macrophage expression in paracancerous tissues. **(C)** Immunofluorescence of S100P and macrophages in cancer tissues. **(D)** Correlation map of S100P and macrophage expression in cancer tissue. **(E)** Correlation map of expression of S100P and NK cells in paracancerous tissues. **(F)** Correlation map of expression of S100P and NK cells in cancer tissues. **(G)** Correlation map of BTK and macrophage expression in paracancerous tissues. **(H)** Correlation map of BTK and macrophage expression in cancer tissue. **(I)** Correlation map of expression of BTK and NK cells in paracancerous tissues. **(J)** Correlation map of expression of BTK and NK cells in cancer tissues.

To verify the accuracy of the results of TCGA and GEO data sets (Supplementary Figures 4A–C), we collected clinical samples from three patients with LUAD. The correlation between the expression of S100P and BTK gene in macrophages and NK cells was analyzed by immunofluorescence. It was found that S100P gene expression was localized on cancer cells and higher in tumor than in paracancerous tissues (Figures 9A,C). The expression of S100P was positively correlated with M2 macrophages (Figure 9B). In tumor tissues, S100P was negatively correlated with M1 macrophages (Figure 9D, *p* = 0.012) and positively with M2 macrophages (Figure 9D, *p* = 0.033). It can be seen that S100P may be closely related to the transformation of macrophages in the order of number list 9→8→5→2 and 9→6→1→2, and it was found that along with the increase of S100P, the number of M2 macrophages increases (Figure 9C). In NK cells, S100P was not localized, but highly expressed in tumor tissues (Supplementary Figures 7A–D). There were not correlations between S100P and NK either in paracancerous tissues (Figure 9E, *p* = 0.214) or tumor tissues (Figure 9F, *p* = 0.808). It can be concluded that the expression of S100P is closely related to the infiltration of M2 macrophages.

BTK in paracancerous tissues was significantly higher than that in tumor tissues (Supplementary Figures 8A–D), which was consistent with the results in the TCGA and GEO (Supplementary Figure 4A). BTK is mainly located on macrophages (Supplementary Figures 8A–D), though not related to any types of TAM (Figures 9G,H). BTK was not localized on NK cells (Supplementary Figures 9A–D). Between NK cells and BTK levels, no correlation was found in paracancerous tissues (Figure 9I, *p* = 0.479), but there was a negative correlation (Figure 9J, *p* = 0.0003) in cancer tissues. These data suggest that lower BTK in tumor tissues is possibly related to the infiltration of NK cells.

### S100P Suppression Inhibits M2 Macrophages Polarization

**FIGURE 10:**
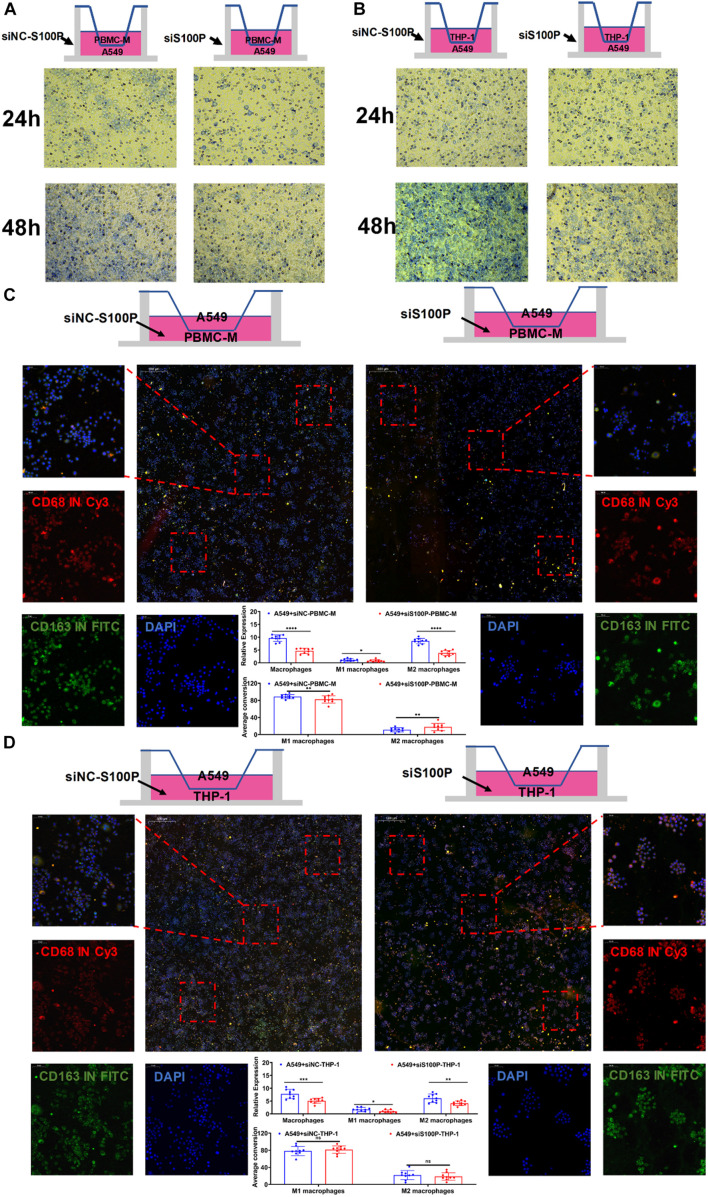
Primary macrophage culture and siRNA transfection. **(A)** Co-culture of PBMC-M after treatment of A549 cells with siNC-S100P and siRNA-S100P. **(B)** Co-culture of THP-1 after treatment of A549 cells with siNC-S100P and siRNA-S100P. **(C)** Effect of A549 cells knocking down S100P on the transformation of PBMC-M cells. **(D)** Effect of A549 cells knocking down S100P on the transformation of THP-1 cells. *p < 0.05, **p < 0.01, ***p < 0.001, ****p < 0.0001.

The above IF assays *in situ* showed that S100P was closely related to the transformation and migration of TAM in tumor tissue. To further verify the relationship between S100P and macrophage polarization, we designed a loss of function assay by transfecting siRNA_S100P to A549 cells in co-culture with primary or inducible human macrophages (Figure 10). Twelve hours after transfection with siRNA_S100P or siRNA_NC, A549 cells were co-cultured with PBMC-M and THP-1 cells induced by PMA (180 ng/ml) for 24 h, respectively. Cell migration was detected by crystal violet staining and transformation of macrophages to M1 or M2 by IF labeling after co-culture for 48 h. We found that cell migration was inhibited by S100P knockdown in both PBMC-M and THP-1 cells in a time-dependent manner (Figures 10A,B). Similarly, S100P knockdown inhibited the survival of PBMC-M (Figure 10C) and THP-1 (Figure 10D). Interestingly, following S100P downregulation, A549 also inhibited the transformation into M2 in primary PBMC-M (Figure 10C), while this was not observed in THP-1 cells (Figure 10D).

## Discussion

The immune status of TME is closely related to the prognosis of LUAD patients. However, the composition of TME is very complex, including highly heterogeneous tumor stromal cells and dozens of infiltrating immune cells, expressing thousands of DEGs. The relationship between these factors is not fully elucidated. Identification of key genes that can represent the immune status of TME could be a promising approach for predicting the prognosis of LUAD. In this study, we identified three key IRGs – BTK, Cd1c and S100P – from the TCGA database for assessing the immune status of TME and predicting the prognosis of LUAD. We found that the three key genes are specifically expressed in different tumor or tumor-associated cells and affect the prognosis and progression of LUAD patients by directly or indirectly regulating tumor-associated immunocytes (Supplementary Figure 1).

The gene signature of LUAD can be used to predict the outcome of clinical treatment (Jiawei et al., 2020). However, the cellular origin of this gene signature is unclear, because both stromal cells and immune cells in TME express DEGs (Lambrechts et al., 2018; Camara et al., 2020). We used immune score and stromal score to partially represent the gene signature of stromal cells and immune cells (Li et al., 2020; Zhang et al., 2020). It was found that OS of 497 LUAD patients from TCGA was significantly positively correlated with immune score (Figure 1), suggesting that immune status is particularly important for the clinical outcome of LUAD patients (Bi et al., 2020). At the same time, immune score and stromal score were negatively correlated with clinical stages (Supplementary Figure 2), suggesting that immune and stromal components in TME may be involved in the development and metastasis of LUAD (Ge et al., 2019; Levayer, 2020). In accordance with this, we found 741 common DEGs in immune and matrix components. GO and KEGG analysis showed that these genes were mainly enriched in immune regulatory pathways (Figure 2).

The immune status of TME depends on the characteristic expression of IRGs, which can be used to predict the prognosis of LUAD patients. We found 93 IRGs that are characteristically expressed in LUAD, whose functions are mainly enriched in leukocyte proliferation, lymphocyte proliferation, monocyte proliferation, and immune-related activities (Supplementary Figure 3). Cox and Lasso analyses showed that 26 IRGs were associated with the prognosis of LUAD patients, of which BTK, Cd1c, and S100P were the most closely related (Figure 3). Multivariate regression analysis of data from GEO and TCGA showed that IRS based on these three genes could be an independent predictor for clinical outcome in patients with LUAD (Table 3). These results demonstrated the possibility of the development of a specific, sensitive, and cost-effective biomarker assay for the evaluation of immune targeted therapy in patients with LUAD.

We further studied the effects of the three genes on regulating the immune status of TME. We analyzed the abundance of TIC in LUAD tissues and made comparison between LUAD and normal tissues (Figure 7). We found that the cell types and cell amounts in tumor tissues are significantly different from normal tissues. Macrophages and NK cells found in the tumor tissues were significantly correlated with the survival rate of LUAD patients (Figure 7).

BTK is a tyrosine kinase, which belongs to the Tec kinase family and plays an important role in the proliferation and differentiation of B cells (Good et al., 2021; Kim, 2019). However, BTK inhibitors have been extended to treat some of the solid tumors, such as non-small cell lung cancer, breast cancer, and pancreatic cancer (Zhang B. et al., 2019; Metzler et al., 2020), suggesting that BTK may affect other immune cells in various TMEs. In accordance with this, TCGA data analysis showed that there was a negative correlation between BTK expression and NK cell abundance (Figure 8), confirmed by clinical tissue samples using an *in situ* labeling experiment (Supplementary Figure 9). Unexpectedly, the expression profile data showed that the expression of mRNA and protein of BTK was inhibited in LUAD tumor tissue (Figure 6 and Supplementary Figure 5), and the down-regulation of BTK was associated with poor survival, which seemed to contradict the strategy of BTK targeted therapy (Pang et al., 2020; Ramalingam et al., 2020). We speculate that the effect of BTK on the immune status of TME depends on its tissue distribution. Immunofluorescence assays of tissue sections showed that BTK was mainly located in macrophages at the junction of cancer nest and stroma, and negatively correlated with the abundance of NK cells (Supplementary Figures 9C,D), suggesting that BTK promotes tumor progression by promoting local immunosuppression. As a specific marker of dendritic cells, Cd1c has the function of regulating antigen presentation of dendritic cells and improving TME (Yuan et al., 2019; Gherardin et al., 2021). We found that both mRNA and protein expression of *Cd1c* were down-regulated in LUAD tumor tissues (Supplementary Figures 4, 5), while the down-regulation of Cd1c was associated with poor survival (Supplementary Figure 4). This may be due to the up-regulation of the function of dendritic cells by Cd1c in TME of patients with non-small cell lung cancer (Bol et al., 2019). S100P is highly expressed in a variety of tumor cells and is a biomarker for early diagnosis of tumors. S100P inhibitors have been used in targeted therapy of LUAD (Tan et al., 2019; Kazakov et al., 2020). Consistent with this, we analyzed the TCGA and GEPIA databases and found that S100P was highly expressed in LUAD tumor tissues (Supplementary Figure 4), and was significantly negatively correlated with patient survival (Figure 6). However, we found that there was no significant correlation between the staging of LUAD patients and the expression of S100P (Supplementary Figure 5), suggesting that S100P may indirectly promote the growth and metastasis of LUAD by regulating TME. Tumor-associated M2 macrophages are one of the main cell types that promote the formation of tumor immunosuppressive niches (Zhang J. et al., 2019; Pan et al., 2020). Interestingly, we found that high expression of S100P was restricted to tumor cells, and along the direction adjacent to the cancer nest, S100P positive tumor cells and M2 macrophages significantly increased, while M1 macrophages significantly decreased (Figure 9). These results suggest that tumor cells with high expression of S100P may drive the polarization of macrophages in TME toward M2 phenotype (Figure 9). Consistent with the results, cell co-culture assays in vitro showed that S100P knockdown in lung cancer cells could inhibit the migration and M2 polarization of primary or cell line-derived macrophages (Figure 10). The discovery of an S100P-induced tumor immunosuppressive niche may provide a new understanding for targeted therapy based on S100P (Zhang et al., 2021).

These findings highlight an important scientific issue for elucidating the immune landscape of TME and understanding the crosstalk between tumor cells and immune cells in TME. Clinically, we constructed a prognostic prediction model based on key IRGs regulating immune cell subsets in TME. However, due to the complexity of the LUAD microenvironment, more comprehensive analysis is needed to reveal the essential regulatory mechanisms that coordinate immune cells infiltrated in tumors.

## Data Availability Statement

The original contributions presented in the study are included in the article/Supplementary Material, further inquiries can be directed to the corresponding author/s.

## Ethics statement

This study was approved by the Medical Ethics Committee of Anhui University of Science and Technology (No. 20190116).

## Author Contributions

DH and JW contributed to conception and design, and study supervision. QX, RF, JW, JZ, JG, MM, YL, XW, CT, and DH contributed to development of methodology, performance of assays, analysis and interpretation of data, and writing of the manuscript. RF, YX, and DH contributed to review of the manuscript. All authors contributed to the article and approved the submitted version.

## Conflict of Interest

The authors declare that the research was conducted in the absence of any commercial or financial relationships that could be construed as a potential conflict of interest.

## Publisher’s Note

All claims expressed in this article are solely those of the authors and do not necessarily represent those of their affiliated organizations, or those of the publisher, the editors and the reviewers. Any product that may be evaluated in this article, or claim that may be made by its manufacturer, is not guaranteed or endorsed by the publisher.

## Footnotes

1
http://www.ncbi.nlm.nih.gov/geo

2
https://portal.gdc.Cancer.gov/

3
http://www.WebGestalt.org/

4
http://www.STRING-db.org/

5
https://www.ncbi.nlm.nih.gov/geo/query/acc.cgi?acc=GSE31210
