# Supplementary material for: Identification of Key Genes Driving Tumor Associated Macrophage Migration and Polarization Based on Immune Fingerprints of Lung Adenocarcinoma
Source: Front Cell Dev Biol. 2021 Nov 4;9:751800. doi: 10.3389/fcell.2021.751800 (PMC8600368; doi:10.3389/fcell.2021.751800)
Supplement: Supplementary file 1 [file Data_Sheet_1.docx]

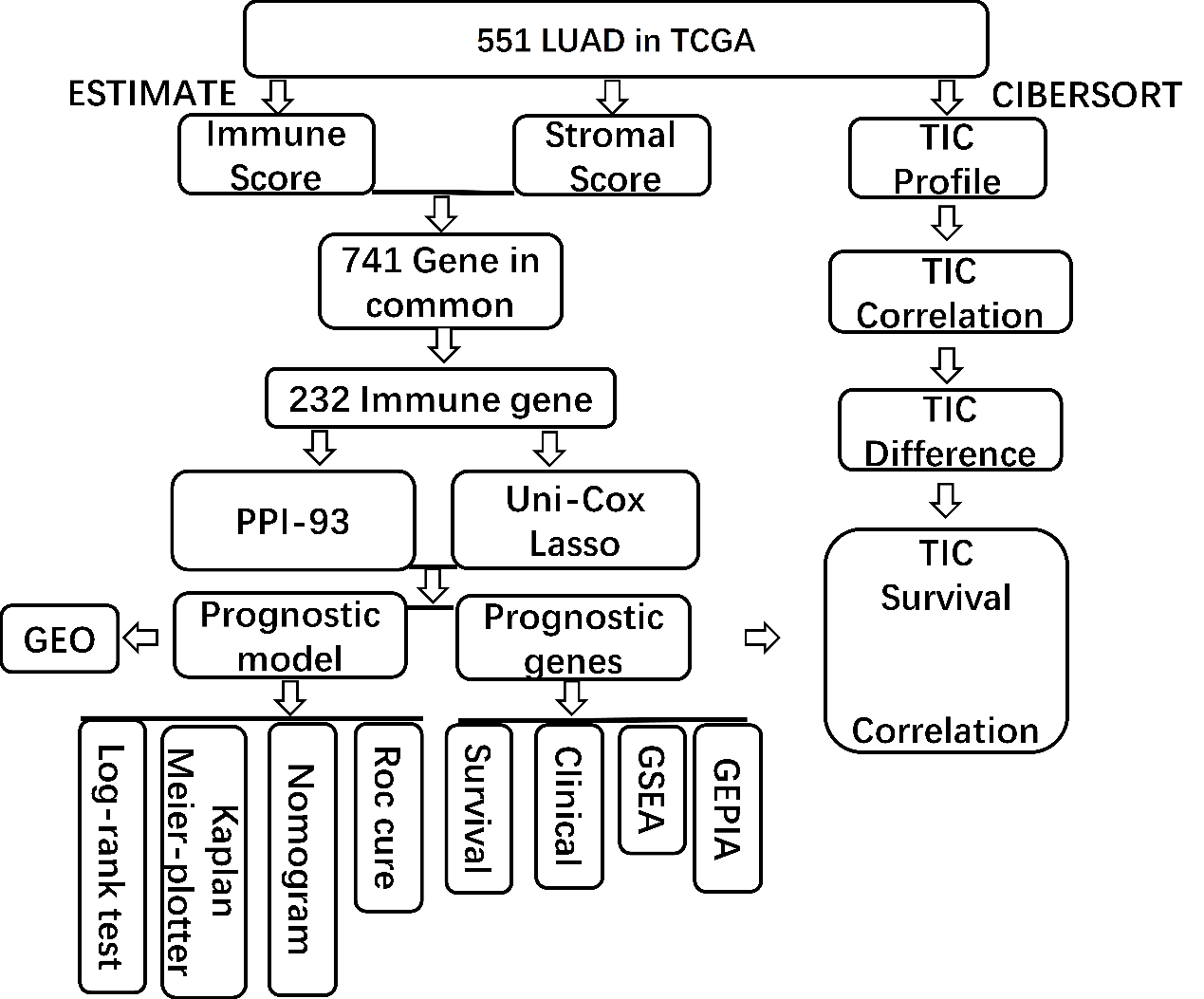


Fig. s1

Fig. s2


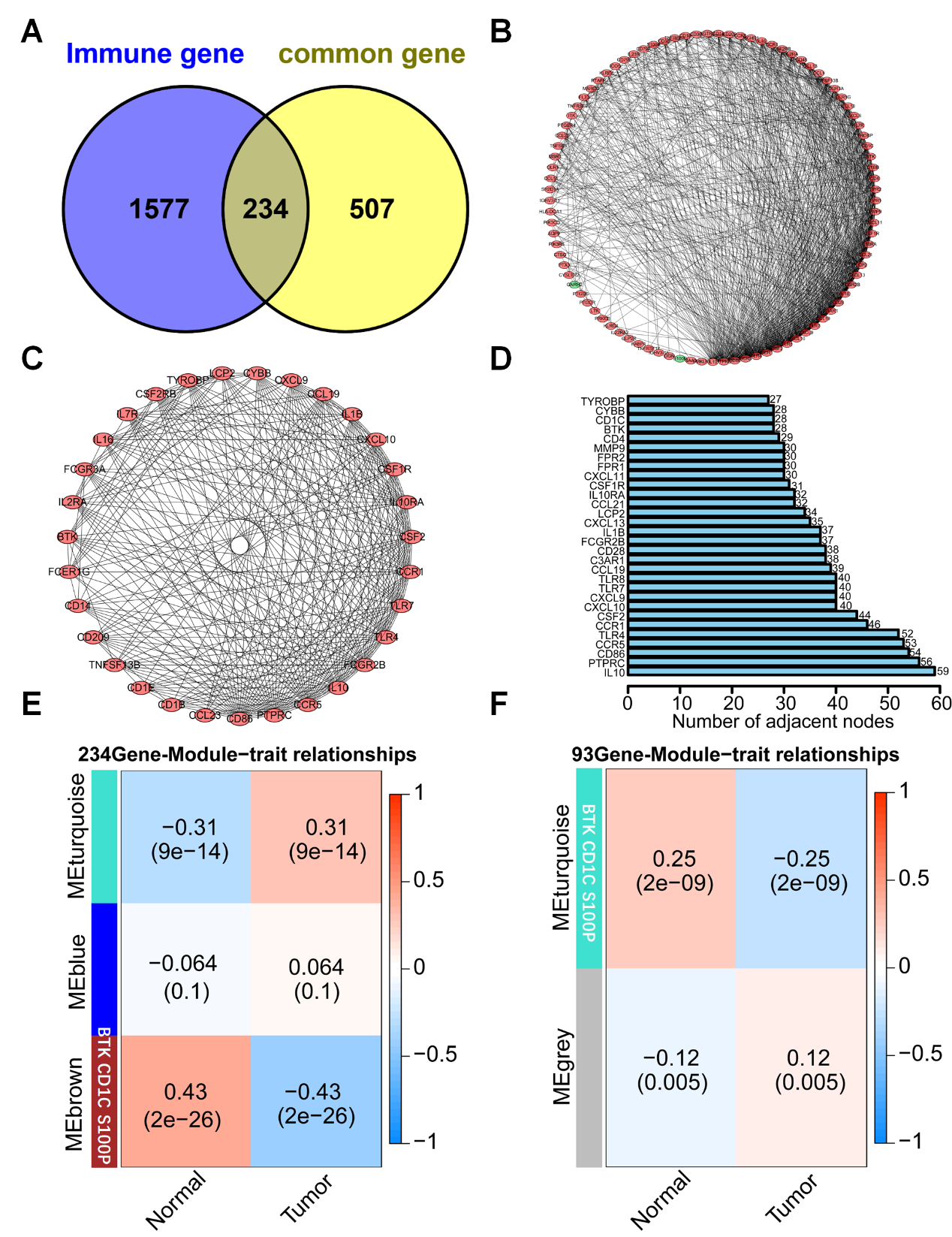


Fig. s3


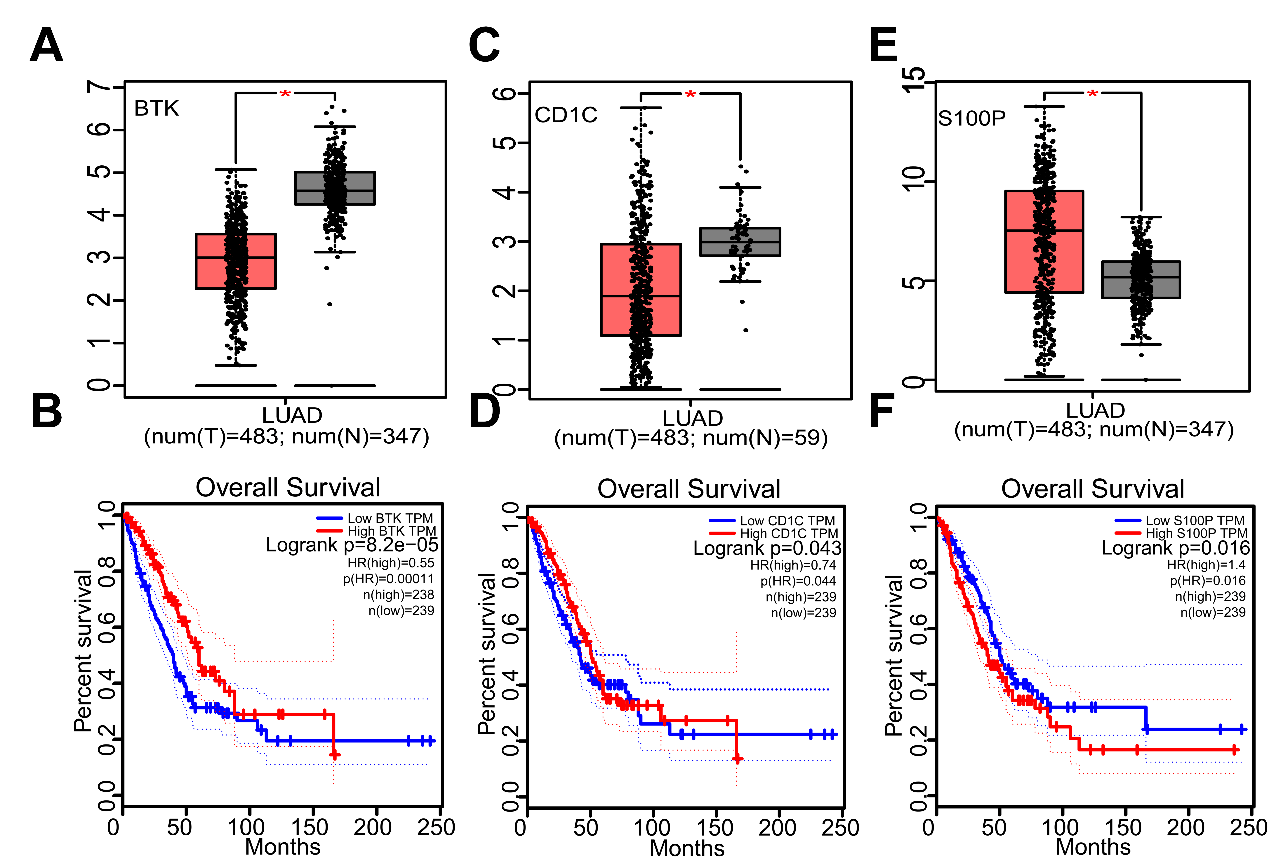


Fig. s4


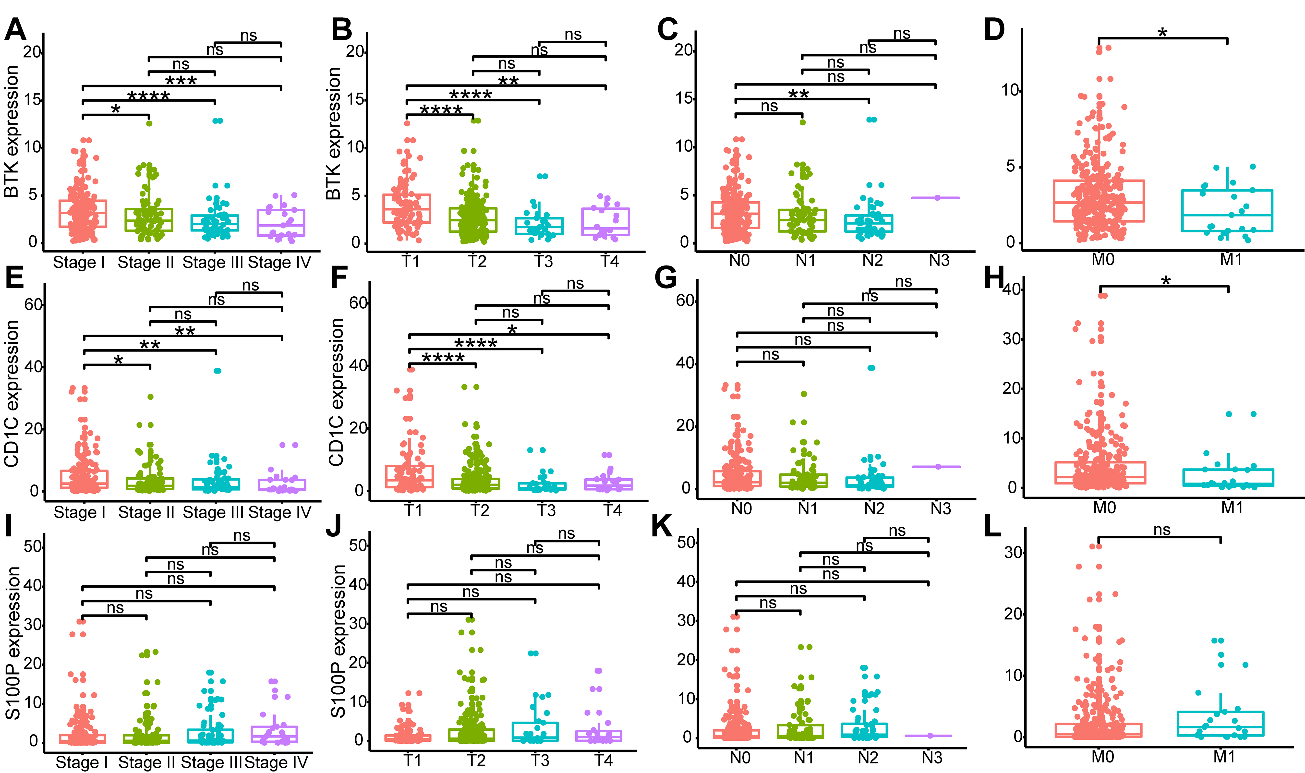
Fig. s5


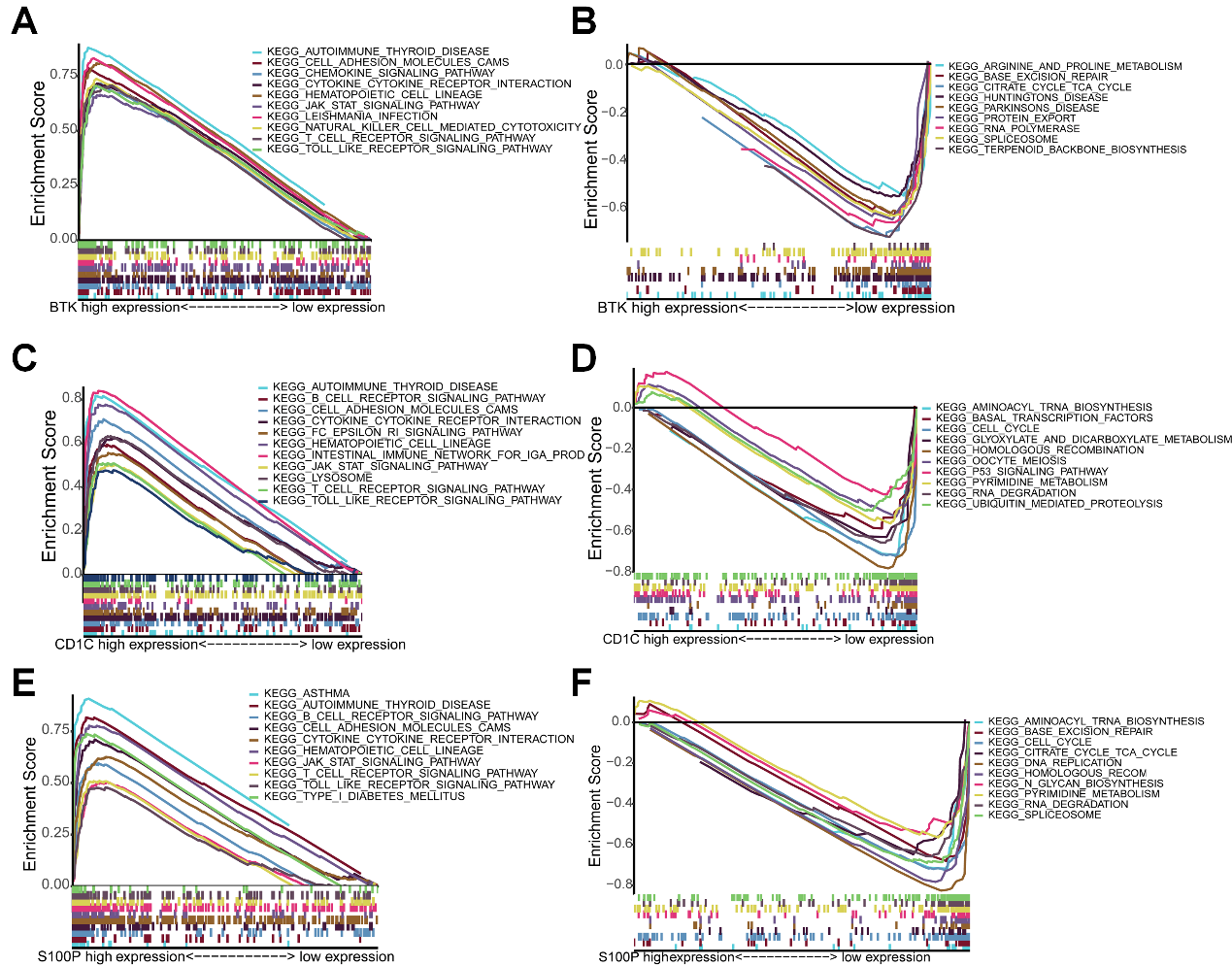


Fig. s6


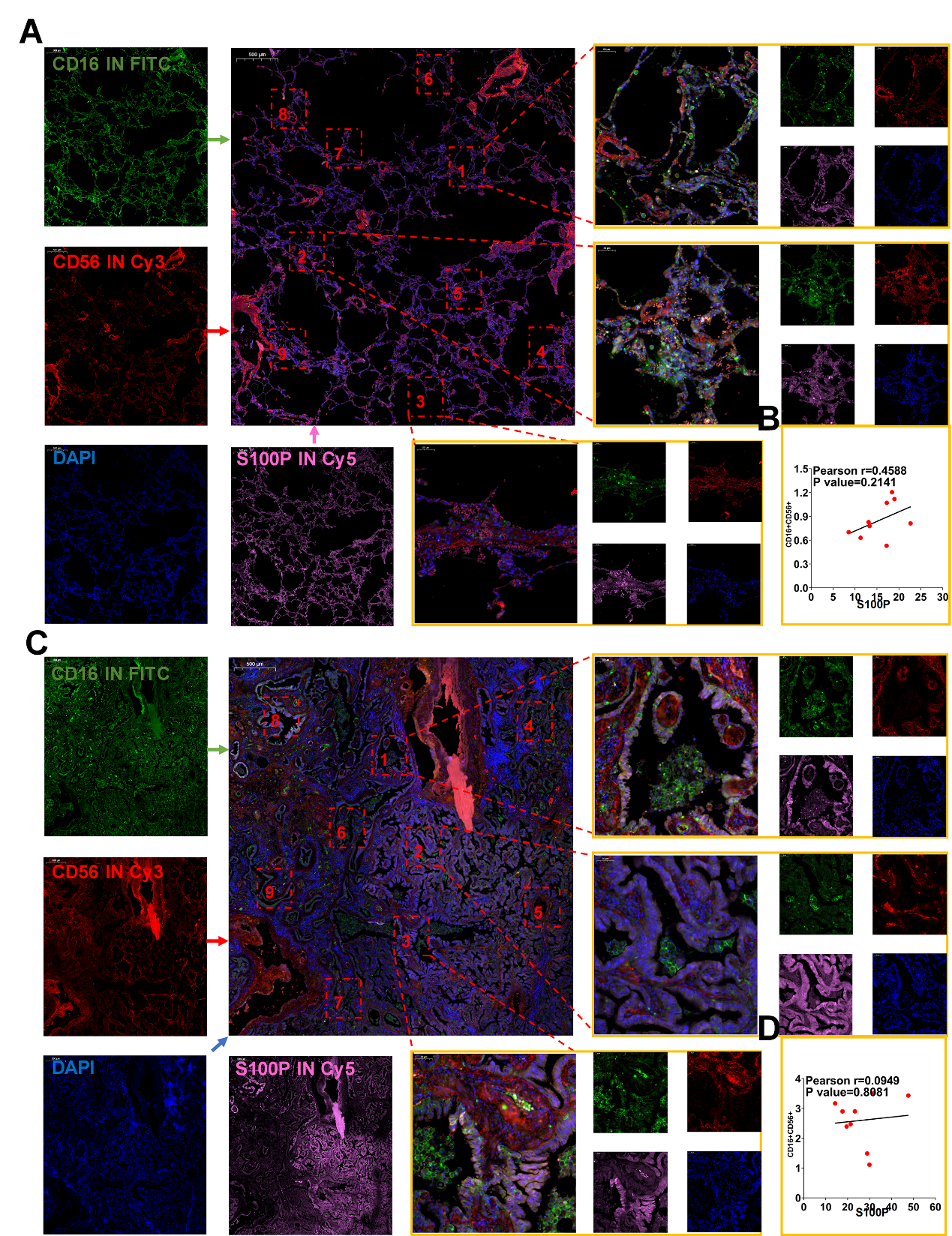


Fig.s7
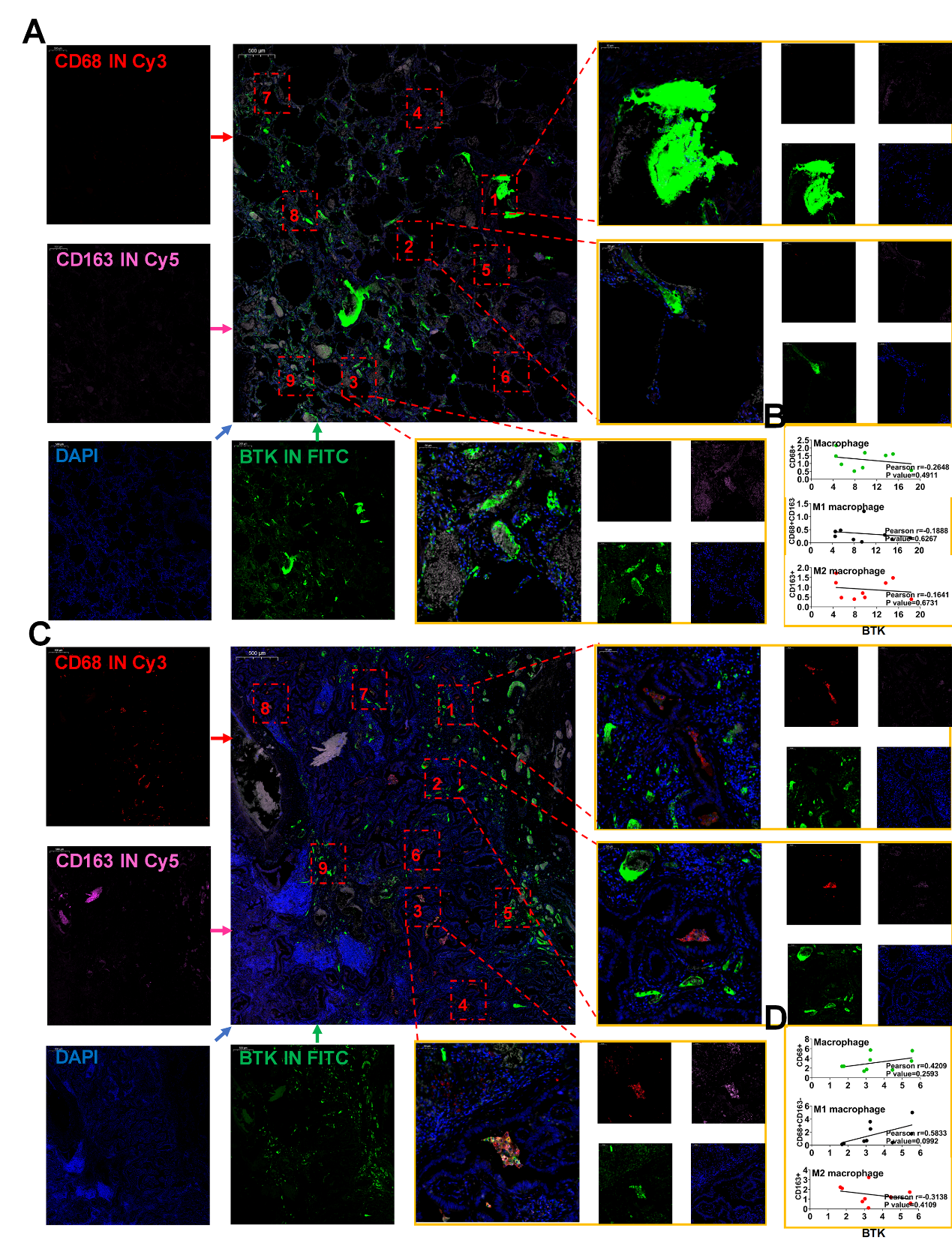
Fig. s8


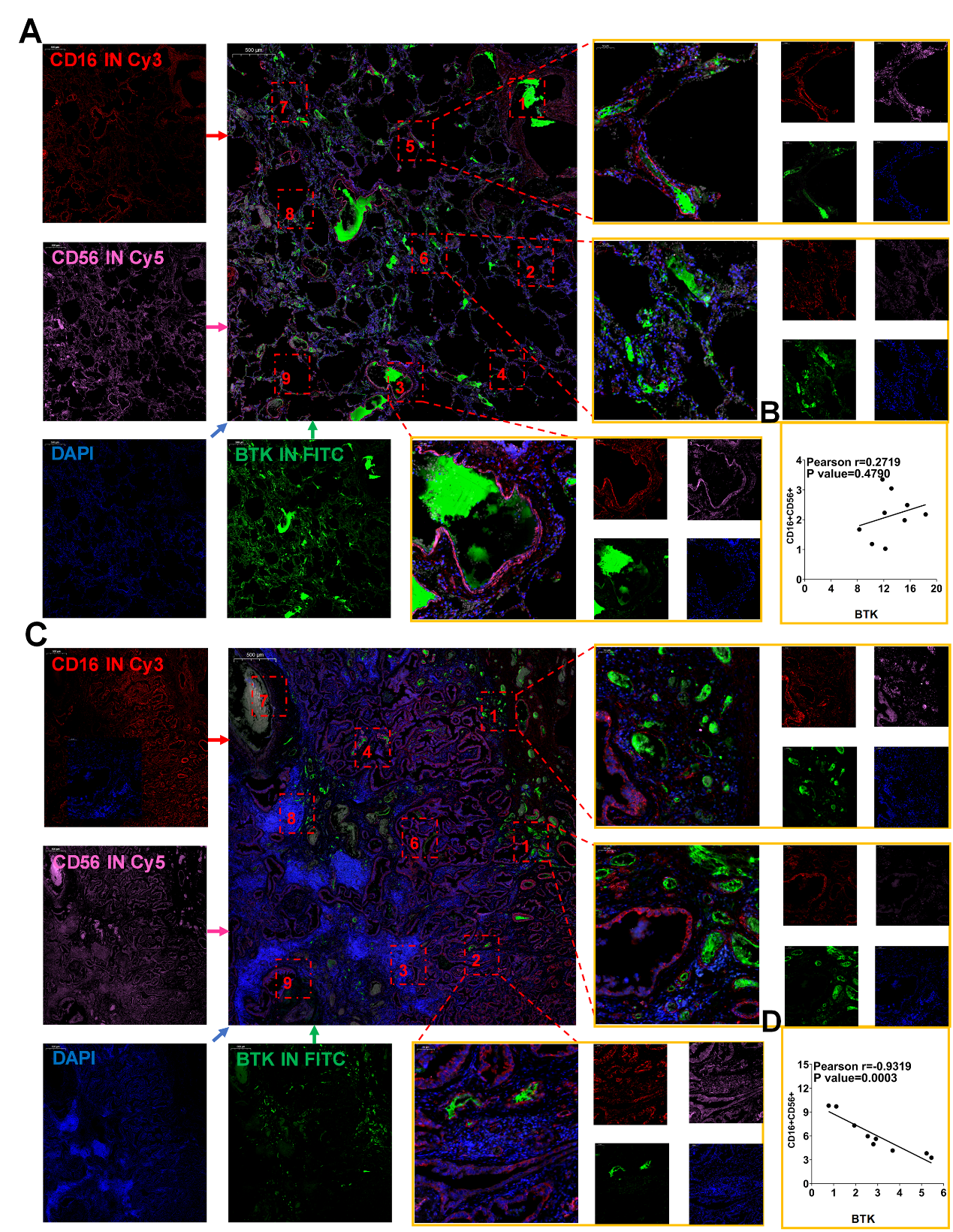


Fig. s9

**LEGENDS**

Fig. s1. Flow chart in construction and validation of 3 prognostic-related IRGs.

Fig. s2. The correlation between immune and stromal score and clinicopathological stage features.

(A-D) The correlation between ESTIMATE score and clinicopathological stage of patients. p<0.05 was statistically significant.

(E-H) The correlation between immune score and clinicopathological stage of patients. p<0.05 was statistically significant.

(I–L) The correlation between stromal score and clinicopathological stage of patients. p<0.05 was statistically significant.

Fig. s3. The analysis of immune genes and PPI network

(A) The Venn diagram of the intersection of IRGs and DEGs

(B) The PPI interaction network of immune genes constructed by Cytoscape

(C) The key module of PPI network constructed by Cytoscape

(D) The top 30 genes in PPI sorted by the number of nodes

(E) Results of WGCNA analysis of 234 key genes in PPI network

(F) Results of WGCNA analysis of 93 key genes in PPI network

Fig. s4. The verification of expression of 3 immune genes and prognosis in network database GEPIA and

(A-B) The verification of expression of BTK in normal and tumor tissues and prognosis of patients in GEPIA

(C-D) The verification of expression of CD1C in normal and tumor tissues and prognosis of patients in GEPIA

(E-F) The verification of expression of S100P in normal and tumor tissues and prognosis of patients in GEPIA

Fig. s5. The correlation between 3 IRGs and clinicopathological stage features

(A-D) The correlation between BTK and clinicopathological stage of patients. p<0.05 is statistically significant

(E-H) The correlation between CD1C and clinicopathological stage of patients. p<0.05 is statistically significant

(I-L) The correlation between S100P and clinicopathological stage of patients. p<0.05 is statistically significant

Fig. s6. The GSEA of 3 immune genes

(A-B) The GSEA of LUAD patients with high- expression and low-expression BTK

(C-D) The GSEA of LUAD patients with high- expression and low-expression CD1C

(E-F) The GSEA of LUAD patients with high- expression and low-expression S100P

Fig. s7.

(A) Immunofluorescence of S100P and NK cells in paracancerous tissues

(B) Correlation map of S100P and NK cells in paracancerous tissues

(C) Immunofluorescence of S100P and NK cells in cancer tissues

(D) Correlation map of S100P and NK cells in cancer tissues

Fig. s8.

(A) Immunofluorescence of BTK and macrophages in paracancerous tissues

(B) Correlation map of expression of S100P and NK cells in paracancerous tissues

(C) Immunofluorescence of BTK and macrophages in cancer tissues

(D) Correlation map of BTK and macrophage expression in cancer tissue

Fig. s9.

(A) Immunofluorescence of BTK and NK cells in paracancerous tissues

(B) Correlation map of BTK and NK cells in paracancerous tissues

(C) Immunofluorescence of BTK and NK cells in cancer tissues

(D) Correlation map of BTK and NK cells in cancer tissues
